# Relaxation Acupressure Reduces Persistent Cancer-Related Fatigue

**DOI:** 10.1155/2011/142913

**Published:** 2010-09-02

**Authors:** Suzanna M. Zick, Sara Alrawi, Gary Merel, Brodie Burris, Ananda Sen, Amie Litzinger, Richard E. Harris

**Affiliations:** ^1^Department of Family Medicine, University of Michigan Medical Center, 715 E. Huron, Suite 2E, Ann Arbor, MI 48104, USA; ^2^The Center for Statistical Consulting and Research, University of Michigan, Ann Arbor, MI 48104, USA; ^3^Department of Anesthesiology, University of Michigan, Ann Arbor, MI 48104, USA

## Abstract

Persistent cancer-related fatigue (PCRF) is a symptom experienced by many cancer survivors. Acupressure offers a potential treatment for PCRF. We investigated if acupressure treatments with opposing actions would result in differential effects on fatigue and examined the effect of different “doses” of acupressure on fatigue. We performed a trial of acupressure in cancer survivors experiencing moderate to severe PCRF. Participants were randomized to one of three treatment groups: relaxation acupressure (RA), high-dose stimulatory acupressure (HIS), and low-dose stimulatory acupressure (LIS). Participants performed acupressure for 12-weeks. Change in fatigue as measured by the Brief Fatigue Inventory (BFI) was our primary outcome. Secondary outcomes were assessment of blinding and compliance to treatment. Fatigue was significantly reduced across all treatment groups (mean ± SD reduction in BFI: RA 4.0 ± 1.5, HIS 2.2 ± 1.6, LIS 2.7 ± 2.2), with significantly greater reductions in the RA group. In an adjusted analysis, RA resulted in significantly less fatigue after controlling for age, cancer type, cancer stage, and cancer treatments. Self-administered RA caused greater reductions in fatigue compared to either HIS or LIS. The magnitude of the reduction in fatigue was clinically relevant and could represent a viable alternative for cancer survivors with PCRF.

## 1. Introduction

Persistent cancer-related fatigue (PCRF), defined as an unusual, constant, subjective sense of tiredness that is not relieved by rest [1], is one of the most common symptoms experienced by cancer survivors and it is often under-diagnosed and often not treated [2]. Rates of significant PCRF in cancer patients range from 30% to 82% within the first five years of diagnosis [3] and as high as 34% five to 10 years after diagnosis [3]. PCRF is associated with decreased quality of life [4–6], decreased sleep quality and/or quantity [5, 6], depression [7], and impaired cognition [8]. Beyond quality of life, subjective reports of low levels of fatigue at diagnosis in breast cancer survivors, for example, predict longer recurrence-free and overall survival even after adjusting for key clinical and sociodemographic confounders [9]. There are few treatment options for PCRF once clear causes for fatigue are identified, for example, anemia. Current treatment options require the availability of a trained practitioner and are also associated with significant costs [10], significant motivation on the part of the patient [10], or have unacceptable side-effects [11]. Consequently, low toxicity, inexpensive, and not difficult to perform treatments, which require minimal instruction by clinic staff would be useful alternatives for treating PCRF. Acupressure, which possesses most of these qualities, could prove a useful treatment for PCRF.

Acupressure is a technique derived from acupuncture, a component of Traditional Chinese Medicine (TCM). During acupressure physical force is applied to acupuncture points by the hand, elbow, or with various devices in an effort to treat disease and reduce symptoms. Acupressure has demonstrated positive effects on sleep quality [12, 13], sleep quantity [14, 15], and depression [15] in cancer patients and other chronically ill populations. Pilot clinical trials have also demonstrated that acupressure and acupuncture can significantly decrease persistent cancer fatigue by as much as 38% [16], although sham acupressure also resulted in similar decreases [16–19]. However, acupuncture in contrast to acupressure can be expensive and requires the availability of a trained professional making it unavailable to some patients. In addition, there is a limit to how many acupressure sessions a person can receive in a given period of time such as a week. This, in turn, limits the “dose” of acupuncture that can be administered. However, patients can administer acupressure on a daily or even several times daily basis without additional cost or need to travel to a practitioner. Despite the potential advantages of acupressure over acupuncture only one previous study has examined acupressure for PCRF [17] and this study was only conducted for two weeks, which may not be enough time to observe clinically significant decreases in fatigue [16, 18, 19]. Also, no previous trials have investigated the effect of dose of treatment on fatigue making it unclear how many weeks, sessions per week, duration of sessions, and so forth, are needed to achieve reductions in fatigue. Thus, we designed a pilot study to examine the effect of longer-term acupressure and how the dose of acupressure impacted PCRF.

Specifically, the purpose of the study was to examine two factors in acupressure treatment: dose and treatment formula. For the former, we examined the effect of 12 weeks of high (HIS) and low (LIS) doses of self-administered stimulating acupressure. For the latter, we compared these two treatment formulas to self-administered relaxation acupressure (RA). Treatment effects were assessed by changes in the severity of chronic fatigue in people diagnosed with cancer who had completed all cancer therapies and who were apparently cancer-free. We hypothesized that the stimulating acupressure treatments (HIS and LIS) would potentially have opposing actions on fatigue when compared to RA. This could occur if the magnitude of the placebo effect was equal in all groups thus allowing for differences in treatment formula to become more apparent. Our previous trial using acupressure to modulate alertness in the classroom served as the basis for this study design [20]. Moreover, we reasoned that the high-dose treatment would be more effective in decreasing fatigue compared to the lower-dose treatment. Secondary objectives were to evaluate the safety, tolerability, adherence, blinding, and beliefs/expectation of participants of the three acupressure treatments.

## 2. Materials and Methods

### 2.1. Participants and Eligibility

The study protocol and all procedures were approved by the University of Michigan Medical School Institutional Review Board and was considered HIPPA compliant. All participants provided written informed consent. People aged 18 years of age and older with a diagnosis of cancer (except for squamous and basal cell carcinomas) who had completed their cancer-related treatments at least 12 weeks prior (except for on-going hormone therapy, which must have been initiated at least three weeks prior to enrollment), were eligible for enrollment. Participants had to have a complaint of persistent, moderate to severe fatigue despite standard treatment (defined as ≥4 on the Brief Fatigue Inventory (BFI)), maintain their typical dietary patterns, especially the use of caffeinated beverages throughout the study, be disease-free, and be acupuncture and acupressure naïve, to be deemed eligible for the study. 

Potential participants were deemed ineligible if they were diagnosed with anemia (defined as hemoglobin levels <12 gm/dL) or receiving treatment for anemia, had any comorbidities likely to cause significant fatigue (e.g., moderate to severe heart failure, hypothyroidism) either currently or before cancer diagnosis, had problems with easy or spontaneous bruising from any cause, for example, bleeding disorders, had nutritional deficiencies (defined by albumin levels <35 g/liter), had a diagnosis of depression and were not receiving active successful treatment for depression or had a HADS depression score of ≥11, had a thyroid disorder (defined as either thyroid stimulating hormone or free T4 lower than the normal range or greater than 2 × s the upper range), had an anticipated survival rate of less than six months, had an initiation, a cessation or change of dose (up to three weeks prior to the study's start) of any chronic medications or dietary supplements or any planned change of chronic medications or dietary supplements during the study, and were pregnant or lactating.

### 2.2. Objectives and Outcomes

This was a pilot clinical study where our primary objective was to test the effect of two intensities of self-administered stimulating acupressure compared to relaxation acupressure on severity of fatigue at 12 weeks as determined by the BFI. The BFI is a validated measure developed at the MD Anderson Cancer Center to screen cancer patients for fatigue. The BFI assesses the severity of and the impact of fatigue on daily functioning in patients with cancer [21]. The BFI is an average of 10 questions where “0” is considered no fatigue or impact on functioning and “10” represents the “worst imaginable fatigue” or “completely interfered” with their daily functioning.

The secondary objectives included: (1) participants compliance to treatment as measured by daily treatment logs [22]; (2) beliefs and expectations of acupressure treatment assessed by questionnaire; (3) safety and tolerability of acupressure treatments determined by reports of adverse events reported and graded per NCI Common Terminology Criteria for Adverse Events (CTCAEs) version 3.0; (4) assessment of blinding, during the 12 week study period.

### 2.3. Randomization, Blinding, and Allocation

Eligible participants were randomized equally to high-dose stimulating acupressure (HIS), low-dose stimulating acupressure (LIS), or relaxation acupressure (RA) groups. The randomization code was computer-generated, and stratified by the three study acupressure practitioners, by the study biostatistician. The randomization list was given to the project manager who had no contact with study participants. For each new participant the project manager chose the next sequential randomization number and linked that with the indicated acupressure treatment and practitioner. The project manager then informed the indicated acupressure practitioner which acupressure treatment to teach to the participant. All study participants as well as all study personnel who assessed outcomes, worked with study data, or administered tests or questionnaires were unaware of the randomization list or treatment assignment except for the project manager and the acupressure practitioners. Participants and study personnel, except the acupressure practitioners and the project manager, were blinded to study assignments.

### 2.4. Recruitment and Screening

Potential participants were identified and referred to us by their oncologist or through the referral of a nurse practitioner that ran a gynecological cancer support group. Participants presented for a screening visit where a history, physical, screening blood work, concomitant medications, and screening questionnaires (BFI and Hospital Anxiety and Depression Scale {HADS}) were obtained. Participants then returned to the study clinic for a baseline visit, within 60 days of the screening visit. At the baseline visit eligible subjects were randomly assigned to self-administer either HIS, LIS, or RA for 12 weeks. Participants in the HIS and RA groups self-administered acupressure for 30 (HIS) or 27 (RA) minutes, respectively, twice per day. The LIS arm was asked to administer acupressure to themselves three times per week for 30 minutes per day.

### 2.5. Intervention

**Figure 1:**
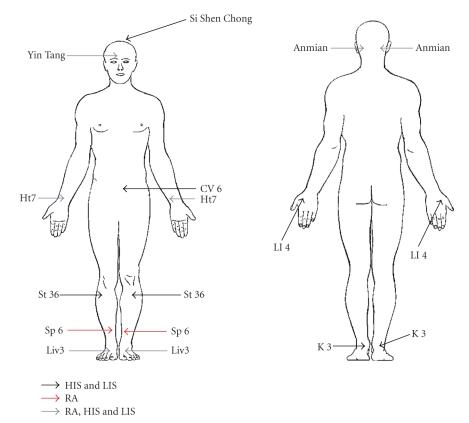
Acupressure point locations. HIS and LIS specific point locations in black: Conception Vessel 6 (CV6), Large Intestine 4 (LI4), Stomach 36 (St36), Kidney 3 (K3), and Si Shen Chong. RA specific point locations in red: Heart 7 (Ht7), Liver 3 (Liv3), Anmian, and Yin Tang. Common point for all groups HIS, LIS, and RA in gray: Spleen 6 (Sp6).

The LIS and HIS acupressure points were identical and consisted of Stomach 36 ({ST36} bilaterally), Spleen 6 ({SP6} bilaterally), Kidney 3 ({KI3} bilaterally), Large intestine 4 ({LI4} bilaterally), Conception vessel 6 (CV6), and Governing vessel 20 (GV20). The relaxation acupressure points consisted of Yin Tang, Anmian (bilaterally), Heart 7 ({HT7} bilaterally), Liver 3 ({LIV3} bilaterally), and Spleen 6 (bilaterally) (see Figure 1). For all three acupressure arms, participants were instructed to make small clockwise circles with their index finger, thumb, or a pencil eraser to stimulate the points for three minutes per point, with sufficient pressure to evoke a “de qi” sensation (i.e., dull ache, tingling, and soreness). Participants were given hands on instructions from one of the three acupressure practitioners at the baseline visit concerning correct pressure and placement of acupressure points. They were also given a diagram with written instructions of the points and a timer to ensure that points were stimulated for the full three minutes. Of note, our protocol used the same amount of pressure (i.e., that required to elicit “de qi”) for all three groups RA, HIS, and LIS. There was no difference in pressure intensities across our three study groups. HIS and LIS were different only in the number of acupressure sessions per week and not different in the intensity of pressure applied at acupuncture points. All participants were asked weekly via phone calls and/or emails if they needed assistance finding any points and encouraged to come back in for retraining if necessary.

Acupressure points were chosen by consensus of the five acupressure practitioners and based on a previous research study examining acupressure for sleepiness [20]. Practitioners had to have been in practice for at least two years actively seeing patients. The acupressure practitioners had received different degrees qualifying them to practice acupressure including a Naturopathic Doctorate (ND), a Masters in TCM or Oriental Medicine and a License of Acupuncture (L.Ac.), or a Diploma in Acupuncture (Dipl. Ac.). Practitioners were asked to choose a set of relaxing points based on a formula to treat insomnia and stimulating acupressure points based on a Western diagnosis of fatigue that could be reasonably reached by participants, that is, not the middle of the back, and not so many points that it would take an excessive amount of time to complete a treatment. 

Participants' beliefs and expectations of acupressure were collected at baseline and at week 12 using a questionnaire. At baseline we asked six questions: (1) What is your impression of acupressure [22]; How much do you want acupressure to reduce your fatigue; (2) how confident are your that applying acupressure to your body will alleviate fatigue; (3) how confident are you in correctly applying acupressure; (4) How successful do you think this treatment would be in alleviating other complaints you may be experiencing; (5) how confident would you be in recommending this treatment to a friend suffering from similar complaints. Similarly at week 12 we asked five related questions: (1) What is your impression of acupressure [22]; How confident are your that applying acupressure to your body did alleviate fatigue; (2) How confident are you that you are correctly applying acupressure; (3) How successful was this treatment in alleviating other complaints you may be experiencing; (4) How confident are you in recommending this treatment to a friend suffering from similar complaints. All questions were answered with a 7-point Lickert scale ranging from, “Not at all Confident or Successful” to “Extremely Confident or Successful.”

Participants also came for a final visit 12 weeks after the baseline appointment where a final BFI, assessment of blinding, and measurement of beliefs and expectations of acupressure were held. Participants were also contacted by email or phone once weekly between the baseline and the final week 12 visit, to enquire about adverse events and to collect BFIs.

### 2.6. Statistical Methods and Sample Size

Baseline characteristics are reported, stratified by treatment group, using means and SDs for continuous variables, and counts and percentages for categorical variables. Balance between treatment groups on baseline characteristics was tested using independent samples *t*-tests for continuous variables and Fisher exact tests for categorical variables. For continuous variables, the assumption of normality was checked using the Shapiro-Wilks test. To investigate a change from baseline in the BFI score, a mixed model analysis was calculated with BFI as the dependent variable and acupressure treatment group, age, type of cancer, stage of cancer, received chemotherapy, received radiation, and week as covariates. 

For examining the group differences in categorical variables including adverse events, assessment of blinding and beliefs and expectations of acupressure Pearson *χ* ^2^ or Fisher exact tests were performed, as appropriate. The association between adherence to treatment in minutes, derived from the daily study log, and change in BFI were calculated using bivariate correlations. Analyses were conducted according to the intention-to-treat principle; however, no imputation was performed for missing values at any of the time points for the BFI score. Data were entered into SPSS Windows version 17 (SPSS, Chicago, IL) and analyzed using SAS version 9. (Cary, NC: SAS Institute Inc.). For all analyses, two-sided tests and a significance level of 0.05 were used. 

We selected a reduction of 3.1 points on the BFI (31.1% [95% CI, 20.6% to 41.5%], improvement) as a clinically meaningful improvement in fatigue severity, based on changes observed in previous acupuncture studies using the BFI to assess differences in cancer fatigue [16]. The study was therefore designed to have 80% power assuming a two-sided alpha level of 0.05 and *n* = 15 participants per treatment group.

## 3. Results

### 3.1. Participants and Toxicity

**Figure 2:**
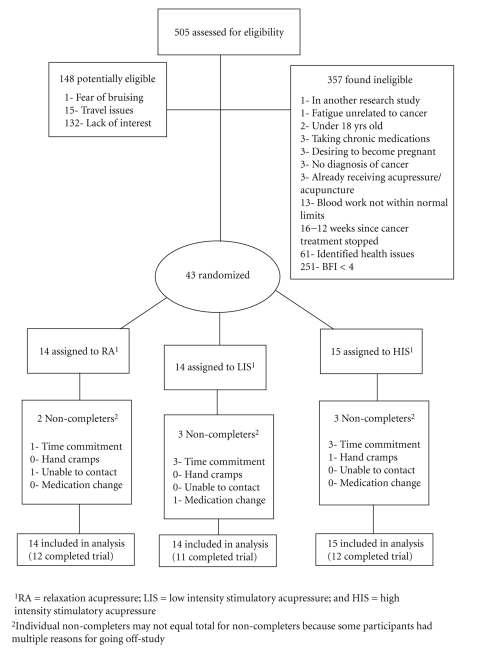
CONSORT diagram of flow of participants through the clinical trial.

We screened 505 people between August, 2008, and April, 2009. Of the 505 people screened, 357 were determined to be ineligible, 148 were potentially eligible but did not complete screening, and 43 met all eligibility criteria and were randomized: 15 to HIS acupressure, 14 to LIS acupressure, and 14 to RA acupressure, for 12 weeks. Figure 2 documents numbers of participants, reasons for exclusions, and reasons for discontinuing the intervention. There was no significant difference between treatment groups for any demographic or clinical characteristic (Table 1). All of the participants but one were women (*N* = 42, 97.6%) with mean age of 54.0 ± 9.0 (range 31–74 years). Over 90% of participants indicated they were Caucasian (*N* = 40, 93.0%) and none of the participant reported being of Hispanic ethnicity. All related toxicities are shown in Table 2. No toxicities greater than NCI Common Toxicity Criteria (v. 3.0) Grade 1 were reported [16, 23]. There was no difference between the groups in terms of total adverse events (*P* = .45) or specific types of adverse events such as musculoskeletal toxicities (*P* = .64). No participants asked for further clarification or training to locate and/or administer their acupressure points.

### 3.2. Change in Fatigue

**Figure 3:**
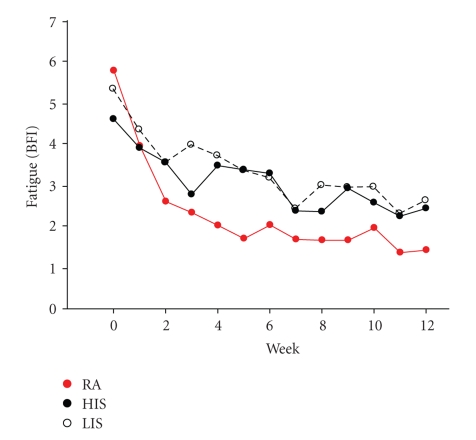
Self-administered relaxation acupressure reduces persistent cancer fatigue. A plot of mean weekly fatigue scores for RA (red circles), HIS (closed black circles), and LIS (open circles) across study weeks demonstrates that RA evokes greater reductions in fatigue scores compared to HIS and LIS.

**Figure 4:**
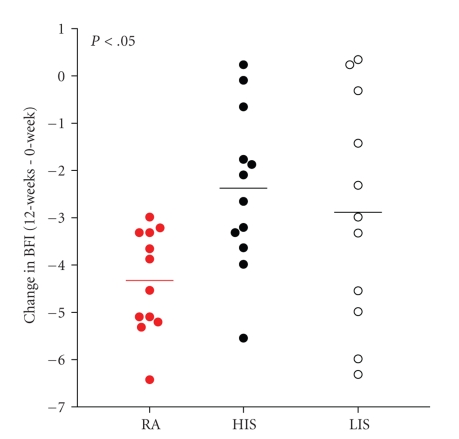
Self-Administered Relaxation Acupressure Engenders Greater Reductions in Fatigue than Stimulation Acupressure. Scatter plot of individual participant BFI change scores (week 12— week 0) indicate greater reductions for RA (red circles) than HIS (closed black circles) and LIS (open circles).

The change in severity of fatigue as assessed by the BFI was significantly different across treatment groups, with greater reductions in the relaxation acupressure group (see Figures 3 and 4; mean ± SD reduction in BFI: RA 4.0 ± 1.5, HIS 2.2 ± 1.6, LIS 2.7 ± 2.2; *P* = .027). These changes represent a mean decrease of fatigue from baseline of 44.8 ± 35.9% in the HIS group, 49.5 ± 35.2% in the LIS group and 70.5 ± 23.4% in the RA group. In a linear regression model with the change in BFI as the dependent variable, the group difference remained significant after adjusting for age, cancer type, cancer stage, and cancer treatments with radiation, chemotherapy, and surgery (*P* = .013). 

The majority of the change in BFI was observed during the first four weeks of acupressure treatment regardless of study group (Figure 3). The mean BFI (all groups) was 5.8 at baseline decreasing to 3.5 at week 4, a 3.3 point drop. The decrease in BFI between weeks 4 to 12 was only 0.9 points from 3.5 to 2.6. Similar patterns were observed in the separate study arms with participants who were randomized to HIS observing a mean decrease of BFI from 4.6 to 3.3; LIS 5.3 to 3.5; RA 5.8 to 2.3, between baseline and week 4. Notably, the small additional decrease in BFI was observed between weeks 4 and 7, while after week 7 mean BFI stayed approximately the same until the end of the study (see Figure 3). 

We defined a responder to acupressure as a person who experiences at least a 31% (based on a 3.1 point decrease on the BFI being considered clinically significant) decrease in fatigue from baseline. Although not statistically significant (*P* = .155), there was a tendency for the study participants in the RA group to be responders (92.9%, *n* = 13), compared to participants in the LIS (66.7%, *n* = 10) or HIS groups (64.3%, *n* = 9).

### 3.3. Blinding and Adherence

Participants were blinded to their acupressure treatment group (*P* = .62). However, in contrast to blinding, there were differences in adherence between the three treatment groups. There was a trend for LIS participants (who were asked to do acupressure only three times weekly versus seven times weekly in the RA and HIS groups) to perform a greater percentage of their treatments compared to either the RA or HIS groups (mean ± SD: LIS 82 ± 30%, RA 70 ± 23%, HIS 50 ± 35%; *P* = .08). Further, across all groups, greater time spent performing acupressure was associated with greater reductions in fatigue (*r* = −0.39; *P* = .037).

### 3.4. Beliefs and Expectations

Across all groups, there was no significant relationship between the belief and expectation that acupressure would help alleviate fatigue and the reduction in BFI at week 12 from baseline for any of the six questions asked on the baseline survey (*P* values ranged from 0.22 to 0.98). The baseline measure of confidence of acupressure in alleviating fatigue was not different across groups (*P* = .10). Most participants were moderately confident that applying acupressure would alleviate their fatigue (RA = 64%; LIS = 29%; HIS = 27%). In a general linear model across groups, with week 12 BFI as the dependent variable and week 0 BFI as a covariate, confidence in acupuncture alleviating fatigue trended towards significance (*P* = .08). At week 12 across groups, there was no significant relationship between confidence in acupressure in alleviating fatigue and actual changes in BFI (*P* = .47).

### 3.5. Discussion

Self-administered relaxation acupressure caused greater reductions in fatigue when compared to either high- or low-dose stimulatory acupressure. This effect was not modified by relevant clinical or demographic variables. Across groups, these reductions in fatigue were on the order of 45% to 70%, which were clinically relevant and could represent significant improvements in quality of life for cancer survivors. 

We observed a much larger decrease in fatigue compared to other studies examining acupuncture and/or acupressure in cancer patients [16–19]. The largest decrease in fatigue reported in other studies was 38% [16] compared to our smallest decrease of 45% observed in the high dose stimulatory acupressure group. Differences in study population, length of study and duration/frequency of acupressure treatments, acupoint locations, fatigue scales, and use of acupuncture rather than acupressure could all be reasons for the difference in fatigue reduction across studies. Perhaps most obviously, three of the four studies examined acupuncture [16, 18, 19] not acupressure to treat cancer-related fatigue. Also, for instance, while two studies examined cancer patients after the cessation of treatments [16, 17], two other studies examined the effect of acupuncture for treating cancer fatigue during radiation treatments [18, 19]. In these later studies, the negative physiological effects of ongoing radiation may account for the less pronounced effect of acupressure on reducing fatigue compared to patients who have completed treatment. 

Another reason we may have observed a greater reduction in fatigue is duration and/or frequency of the acupressure treatment. In three of the previous studies examining acupuncture for fatigue in cancer patients [16, 18, 19], participants received acupuncture one to two times per week for six weeks, while, in another study, participants received six sessions of acupressure or acupuncture over two weeks [17]. The largest decreases in fatigue of 36% to 38% [16, 17], were observed when 12 to 24 sessions of acupuncture were administered whether over two weeks or six weeks, while only a 19% reduction in fatigue was observed when acupressure was self-administered daily for one minute per point (three minutes total) over two weeks [17]. Less pronounced effects on fatigue in these studies compared to ours may be due to both the increased duration and frequency of acupressure treatments in our study. We observed that at least four weeks were needed to achieve significant effects and seven weeks of treatment to reach a maximum effect. Thus, to have the maximum effect on fatigue participants in our study, needed to perform a minimum of 21 to 49 acupressure treatments over seven weeks (three times per week to one time per day, depending on study arm). In fact, we found that fatigue continued to significantly decrease the more acupressure was performed, regardless of the study group.

Differences between our results and previous acupressure/acupuncture trials for cancer fatigue could also be due to different fatigue measures. Only the study by Vickers et al. used the BFI [16]. Other principal measures of fatigue used in other studies include the Multidimensional Fatigue Inventory (MFI) [17], the Lee Fatigue Scale [18], and the Functional Assessment of Chronic Illness Therapy-Fatigue Subscale (FACIT-F) [19]. All of these fatigue scales are able to capture both physical and psychological aspects of fatigue and are useful for both screening and outcome assessments in fatigue trials [24]. However, neither the FACIT-F nor the Lee fatigue scales have been validated in cancer patients and none of these measures have been tested for their convergence or divergence from one another [24]. Lacking this data, it is difficult to make comparisons across measures, although the BFI and MFI are constructed in a similar manner and produced similar results for acupuncture across other studies [16, 17]. 

Earlier studies enrolled samples that were potentially more diverse than our study sample. For instance, in several previous studies, there were significantly more men [16–18], older patients [16, 18], or participants having a wider range of cancer diagnoses compared to our sample [16–18]. Smaller improvements in fatigue in these studies could imply that acupressure is more effective in certain populations such as younger women. Consequently, the relative homogeneity of our study sample of white women predominantly in their 50's could be one where our participants responded more favorably to acupressure than observed in other studies.

Finally, it is possible that the larger reduction in fatigue we observed in our study is due to our choice of acupoints. Active acupoints chosen in earlier studies [16–19] overlap appreciably with the acupoints we chose for both our LIS and HIS groups. Of the six points in our stimulating acupressure groups, two previous trials used five of the same points (ST36, SP6, KI3, LI4, and CV6) [16, 18, 19], one used four of the same points (ST36, SP6, KI3, and CV6), and one used three of the same points (ST36, SP6, and LI4) [17, 18]. In contrast, only one study included a point in either their true or sham acupoints that overlapped with our RA points (LIV3) with the exception of SP6 which we included in both our stimulating and relaxation groups. This would argue that there may be some specific effect of the relaxation acupoints on fatigue different from or of a larger magnitude than for the stimulating acupoints. One possible mechanism for RA could be through improving sleep quality, as sleep disturbances are positively correlated with persistent fatigue and are a significant predictor of persistent fatigue [25]. While we did not examine sleep parameters in this study, in a separate study in fatigued college students [20], we showed that participants were less alert and more sleepy following relaxation compared to stimulatory acupressure. Of note, the acupoints used in that study were almost identical to the ones we used in the current study in cancer survivors. Further investigation of how different acupressure techniques may diverge or converge in their affect on sleep quality and other key mechanisms of PCRF would help to clarify the specific role of different acupoints.

We also investigated the effect of dose of acupressure on fatigue. Very few studies have assessed the effect of dose on the efficacy of acupuncture or acupressure treatments. This has led researchers to posit that acupuncture studies with null findings could be due to false negative results from inadequate number, length, or duration of treatments, for instance. At least two studies support the idea that fewer sessions per week or shorter length of acupuncture treatment are not as effective at decreasing pain. Harris et al. [21] found that three acupuncture sessions weekly provided more pain relief than one session weekly (*P* = .039). Another research group [26] discovered that the difference between sham and true acupuncture for pain was not evident at eight weeks, but was statistically significant at 14 weeks. We, however, found no difference in fatigue between our high-dose and low-dose stimulating treatments. This was despite the low dose performing only three 30 minute sessions per week compared to the high dose performing two 30 minute sessions daily. Even when decreased adherence in the HIS group was taken into account, the participants were still consistently administering at least one 30 minute session daily, which is appreciably more than the LIS group. Thus, it would appear that the duration in weeks of acupressure, not the frequency or the total number of treatments, were of more importance for decreasing persistent fatigue. Future studies could examine this question in more depth as well as observing how long treatment effects exist after patient stop administering acupressure.

Self-administered acupressure was exceedingly safe and well tolerated with only nine minor transitory adverse events. Moreover, no participant stopped their acupressure or withdrew from the study as a result of these effects. The acupressure treatments also appeared to be an acceptable treatment for cancer survivors. However, it appears to be neither feasible nor desirable to ask participants to perform acupressure more than once daily. Participants randomized to RA and HIS who were asked to perform acupressure twice daily, were less compliant, and in general performed their acupressure only once daily. 

Except for one question, participants' belief and expectations of acupressure did not predict response to acupressure treatment for PCRF. However, the question asked at baseline about confidence in acupuncture alleviating fatigue approached statistical significance (*P* = .08) for predicting change in week 12 BFI. Also, while only approaching significance, more people who were randomized to the RA arm indicated that they were confident in acupuncture alleviating their fatigue compared to either the LIS or HIS arms (RA = 64%; LIS = 29%; HIS = 27%; *P* = .10). Consequently, confidence in acupressure could be an important predictor and/or mediator of its ability to alleviate fatigue, with those who are more confident receiving a larger benefit from the treatment. Other studies examining patients' expectations of and confidence in acupressure or acupuncture show mixed results [24–29] with some studies showing a significant association between reduction in pain and confidence in acupressure and/or acupuncture [27–31] and others finding no association [32]. Unfortunately, acupressure and acupuncture studies examining the effect of belief and confidence in the treatments have focused largely on pain, not fatigue, as an outcome. As such, it is difficult to draw comparisons between our study's results concerning belief and expectations and other acupuncture or acupressure studies. Clearly, this is an area that requires further research into what extent and how confidence in acupressure affects its efficacy.

This study had several limitations. As this study was conceived as a pilot and feasibility trial, we had only a small sample size of 43 participants. Larger studies in cancer survivors would be needed to confirm and compare results from this trial. Also, our study sample lacked variability with the majority of participants being white women diagnosed with breast, ovarian, or endometrial cancer. As such, the results have limited generalizability. Further studies would be needed to investigate the effects of self-administered acupressure for fatigue in other populations such as men, racial or ethnic minorities, other cancer types, and in children. This study also did not examine any mechanism by which acupressure caused decreased fatigue. Future studies would be needed to examine both behavioral and physiological mechanisms to help better understand the full utility of acupressure in the clinical setting.

Along with a greater effect in reducing fatigue, acupressure has several advantages over acupuncture treatments: it can be self-administered with little effort and time on the part of the patient, it is well tolerated, of low-cost, and requires minimal instruction by clinic staff, for example, a nurse. Patients with needle phobias and without severe bleeding disorders or issues with bruising can still benefit from acupressure when acupuncture would be contraindicated.

In summary, self-administered acupressure holds significant potential for being a cost-effective, low-toxicity, self-care treatment for PCRF, one of the most troubling symptoms for cancer survivors. Further research is needed to elucidate the mechanisms behind acupressure's effect on fatigue. In particular, our research indicates that further investigation should focus on the distinct role of the effects of specific acupoints and expectations of acupressure. Other important areas for potential investigation include the role of “dose” and specifically duration of acupressure treatment and differing effects of acupressure in diverse populations.

## Figures and Tables

**Table 1:** Baseline characteristics.

	Relaxation acupressure (*n* = 14)	Low-intensity acupressure (*n* = 14)	High-intensity acupressure (*n* = 15)
*Demographics*			
Sex *n*(%)			
Women	14 (100.0)	13 (93.0)	15 (100.0)
Men	0 (0.0)	1 (7.0)	0 (0.0)
Age (mean years) ± SD	51.5 ± 6.7	54.4 ± 10.0	56.0 ± 9.3
Race *n*(%)			
White	12 (86.0)	13 (93.0)	15 (100.0)

*Clinical characteristics*			
BFI at baseline (mean ± SD)^a^	5.8 ± 1.2	5.3 ± 1.7	4.4 ± 2.0
Confidence in acupressure^b^	9 (64)	4 (29)	4 (27)
Cancer type *n*(%)^c^			
Breast	8 (57.0)	7 (50.0)	9 (60.0)
Uterine	1 (7.0)	2 (14.3)	1 (6.7)
Cervical	1 (7.0)	1 (7.1)	0 (0.0)
Endometrial	3 (2.0)	1 (7.1)	1 (6.7)
Ovarian	1 (7.0)	1 (7.1)	4 (26.6)
Other^d^	1 (7.0)	2 (14.1)	0 (0.0)
Stage of cancer *n* (%)^c^			
Stage 1	9 (64.3)	8 (57.1)	8 (53.0)
Stage 2	4 (28.6)	3 (21.4)	3 (20.0)
Stage 3	0 (0.0)	1 (7.1)	3 (20.0)
Stage 4	0 (0.0)	2 (14.3)	1 (7.0)
Unknown	1 (7.1)	0 (0.0)	0 (0.0)
Time since cancer diagnosis in months (mean ± SD)^e^	37.9 ± 35.3	36.4 ± 47.6	44.6 ± 49.2

*Treatments (were received)* *n* *(%) *			
Surgery	14 (100.0)	13 (93.0)	15 (100.0)
Chemotherapy	7 (50.0)	7 (50.0)	10 (67.0)
Radiation	8 (57.0)	10 (71.0)	10 (67.0)
Immunotherapy	0 (0.0)	0 (0.0)	1 (7.0)
Other	0 (0.0)	1 (7.0)	2 (13.0)

^a^BFI: Brief Fatigue Inventory.

^b^What percentage of participants were at least moderately confident that acupressure would alleviate their fatigue at the baseline visit.

^c^Percentages may not add up to 100% because participants can receive multiple treatments or diagnoses.

^d^Melanoma, colorectal, unknown primary.

^e^“Time since Cancer Diagnosis” was calculated from on-study date and date of diagnosis in months.

**Table 2:** Adverse events by person.

		*N* (%)		
Adverse events	RA (*n* = 14)	LIS (*n* = 14)	HIS (*n* = 15)	*P*-value^a^
Participants with any adverse events	5 (36)	0 (0)	4 (27)	0.45
Musculoskeletal^b^	3 (21)	0 (0)	3 (20)	0.64
Other^c^	2 (14)	0 (0)	1 (7)	0.64

^a^ *P*-values were calculated using Pearson chi-square test.

^b^Musculoskeletal Symptoms include: leg cramps, hand cramps, achiness, osteoarthritis diagnosis, tenderness, and mild bruising.

^c^Other includes: dizziness, hot flashes, and transient sleep issues.

All adverse events were given a grade 1 on the Common Terminology Criteria for Adverse Events v3.0.
